# Continuity in Drinking Water Supply

**DOI:** 10.1371/journal.pmed.1001894

**Published:** 2015-10-27

**Authors:** Clarissa Brocklehurst, Tom Slaymaker

**Affiliations:** 1 Department of Environmental Sciences and Engineering, Gillings School of Global Public Health, The University of North Carolina at Chapel Hill, Chapel Hill, North Carolina, United States of America; 2 Data & Analytics Section/Division of Data, Research and Policy, UNICEF, New York, New York, United States of America

The benefits of having a continuous, piped supply of safe drinking water delivered to household premises are widely recognised. Piped supplies on premises not only reduce the time and effort required to collect water, and thereby increase the amount of water available for personal and domestic needs but also are more likely to provide water that meets required standards for drinking water quality. A recent systematic review of drinking water quality [1] confirmed that piped water supplies are less likely to be contaminated than other types of “improved” sources, such as hand pumps, protected wells, and springs.

However, continuity of piped supplies—that is, the uninterrupted supply of water—and the associated health impacts, are under-studied. Two papers in this issue of *PLOS Medicine* each shed light on a particular aspect of continuity and are a very welcome addition to the literature.

Jeandron and colleagues [2] show that in the city of Uvira, in the Democratic Republic of the Congo, there was a significant relationship between interruptions in the piped water supply and cases of suspected cholera. This association was seen even though many in the city used piped services indirectly (that is, they did not have household connections and used water from standposts and shared connections). The interruptions in the piped water supply thus represented a lost opportunity to protect a cholera-vulnerable population.

In Hubli-Dharwad, in the state of Karnataka in India, Ercumen and colleagues [3] show that while upgrading the piped supply to provide continuous service for 10% of the population was associated with a notable reduction in typhoid among the poor in the beneficiary group, they did not find the expected decrease in diarrhoeal diseases. The paper suggests a number of possible reasons, including unhygienic storage of water due to the use of yard taps rather than taps in the house; the perceived threat of supply interruptions; heavy contamination of the immediate household environment due to widespread open defecation among children; and the continuing presence of open sewers. It is notable that the research in India found a strong relationship between the continuity of the supply and quality of the water delivered. Among water samples taken from households receiving a continuous supply, less than 1% did not comply with WHO guidelines for drinking water quality, compared with over one-third of samples in households that did not benefit from improvements and still had intermittent supply.

The importance of continuous piped supply to households in order to protect health is clear. This link is not surprising given the engineering reality—that maintaining continuous positive pressure in a piped network and avoiding negative pressure at all costs prevents infiltration of contaminated soil water [4]. Continuous supply also removes the need to store water and makes water readily available for hygiene, such as handwashing.

From a public health policy point of view, ideally, households should have continuous water supply, treated with chlorine, piped directly into the house, with accompanying improvements in the sanitary environment. For this reason, a new benchmark has been proposed to be included in the indicators used for monitoring progress towards the Sustainable Development Goals adopted by the United Nations (UN) General Assembly in September 2015.

The new term “safely managed drinking water services” is proposed as the highest level of service countries should aspire to reach, and refers to a source of drinking water that is on premises, available when needed (that is, in the case of piped supplies, continuous), and free of faecal and priority chemical contamination. This represents a significant step up from the highest level of service used in monitoring during the period of the Millennium Development Goals: “improved drinking-water source,” which did not include measures of distance to the home, quality or continuity of water supplied, and included off-premises sources such as hand pumps, dug wells, and springs.

Reaching this new benchmark at a global scale is undoubtedly ambitious. However, monitoring data [5] show it is not impossible. During the last 25 years, coverage of piped water on premises increased from 44% to 58% globally, so now well over half the world’s population benefits from this level of service. In those countries designated as developing countries by the UN, the use of piped water on premises has grown even faster, from 31% in 1990 to an estimated 49% in 2015, representing an additional 1.7 billion people with piped water connections. Progress in some regions has been even faster, and in Eastern Asia the number of people with piped water on premises skyrocketed from 30% to 74%, mostly as a result of rapid increases in China.

Continuous, treated, piped supply to every household should be our ambition, even though achieving it may be many years in the future, and in many settings, interim arrangements with lower levels of service are going to be the reality. However, many countries have decided providing piped supply is worth the investment, and innovations are being rolled out in many places to find robust, inexpensive ways to provide this service in an affordable way. The studies in this issue of *PLOS Medicine* highlight the importance of continuous piped water supply, but also the pitfalls. Interruptions in the supply can negate the possible health benefits, and installing piped water without addressing other environmental concerns may not deliver the hoped-for health benefits. It must not be forgotten that piped water supplies require more than just infrastructure; good governance and competent management are needed to ensure continuous service.
